# Clinical utility of a host-protein test for suspected infection in the pediatric emergency department: a pragmatic pre-/post-implementation study

**DOI:** 10.3389/fped.2026.1803886

**Published:** 2026-05-15

**Authors:** Vered Nir, Vered Schichter Konfino, Naama Kuchinski Cohen, Esther Levy, Noa Kremer, Yosef Or Shamia, Amir Nakar, Boris Lebedenko, Jeroen Stas, Tanya M. Gottlieb, Ma’anit Shapira, Michal Stein, Adi Klein

**Affiliations:** 1 Pediatrics Department, Hillel Yaffe Medical Center, Hadera, Israel; 2 Rappaport Faculty of Medicine, Technion Institute of Technology, Haifa, Israel; 3 Pediatrics Emergency Department, Hillel Yaffe Medical Center, Hadera, Israel; 4 Clinical Laboratory Division, Hillel Yaffe Medical Center, Hadera, Israel; 5 Pediatrics Department, Carmel Medical Center, Haifa, Israel; 6 MeMed, Tirat Carmel, Israel; 7 Pediatric Infectious Diseases Unit, Sheba Medical Center, Edmond and Lily Safra Children’s Hospital, Tel-Hashomer, Israel; 8 Gray Faculty of Medicine, Tel Aviv University, Tel Aviv, Israel

**Keywords:** antibiotic stewardship, biomarkers, emergency department, host-response testing, MeMed BV

## Abstract

**Background:**

Identifying infectious etiology in pediatric emergency medicine can be challenging, often leading to antibiotic misuse. MeMed BV (MMBV) is a host-protein test that accurately differentiates bacterial from viral infections, but real-world utility data in children are limited.

**Methods:**

We performed a single-center retrospective study of preschool-aged children enrolled in two pragmatic cohort studies, where MMBV was ordered at physician discretion. In the “pre” study, MMBV results were not available (standard of care, SC arm), whereas in the “post” study, MMBV results were available in a timely manner (MMBV arm). The primary endpoint was change in antibiotic prescribing rate among outpatients with viral MMBV results. Secondary endpoints included prescribing rate changes in a lower respiratory tract infection (LRTI) subcohort and changes in hospital length of stay (LOS).

**Results:**

The study cohort included 1,022 children in the SC arm and 474 in the MMBV arm. Antibiotic prescribing in outpatients with viral MMBV results decreased from 20.3% in the SC arm to 7.5% in the MMBV arm (*p* = 0.007). Prescribing increased in outpatients with bacterial MMBV results (*p* = 0.111), resulting in a net decline across outpatients in the SC versus MMBV arms (25.0% vs. 16.8%; *p* = 0.071). In the LRTI subcohort, there was a prescribing decreased (84.0% to 67.9%; *p* = 0.002) irrespective of patient disposition. Despite more severe clinical presentation, patients in the MMBV arm had a shorter length of stay (LOS) compared with patients in the SC arm (3.1 ± 1.9 vs. 3.6 ± 1.9 days; *p* < 0.001).

**Conclusions:**

Implementation of MMBV in routine pediatric emergency care was associated with optimized antibiotic use and shorter hospital LOS.

## 1 Introduction

Children presenting with fever and suspected acute infections to the hospital require prompt clinical decision-making regarding antimicrobial use and care escalation. Clinical symptoms and vital signs alone are often insufficient to inform appropriate antibiotic use due to the overlapping symptoms of bacterial and viral infections (1). A conservative approach to prescribing antibiotics involves weighing the benefit of not prescribing antibiotics versus the risk of missing potentially serious bacterial infections. However, clinical uncertainty leads to unnecessary antibiotic overuse, which contributes to the growing problem of antimicrobial resistance and avoidable adverse events (2, 3). Clinical uncertainty also increases the risk of hospital admission and length of stay, thereby escalating costs (4, 5). The decision-making process is further complicated by external pressures, such as those from parents (6–8). In summary, infectious etiology—a critical factor in deciding the need for antibiotics—does not adequately guide actual clinical practice.

A range of diagnostic tools exist to inform antibiotic decisions. The utility of single biomarkers, such as CRP or procalcitonin, remains disputed, and current stewardship guidelines do not recommend their use for initiating antibiotics in ED patients with respiratory tract infection (RTIs) (9–13). Pathogen-directed tests, widely adopted during the COVID-19 era, are constrained in their utility because viral detection does not rule out a bacterial co-infection, and more often than not, no pathogen is detected (14–16). Systematic reviews show that rapid respiratory viral testing does not aid in appropriately targeting antibiotic prescription, underscoring the need for novel diagnostic approaches (17, 18).

Host-response panels for differentiating between bacterial and viral infections are a new category of omics-based diagnostics that map the patient’s immune response to determine infectious etiology (1). MeMed BV (MMBV)—cleared for use in adults and children—measures three host-protein biomarkers (TRAIL, IP-10, and CRP) and provides a bacterial (including co-infection) likelihood score (19). MMBV has consistently yielded high diagnostic accuracy across multiple prospective validation studies in urgent care, emergency department, and inpatient settings, with AUC values ≥0.9 and high negative predictive values for ruling out bacterial infection (19–23). Furthermore, controlled use of MMBV in pediatric emergency departments has been associated with reduced unnecessary antibiotic prescribing in low-risk children under 5 years of age, as well as reduced multiplex respiratory PCR testing (24). In adults with suspected lower RTI (LRTI), a recent randomized controlled trial revealed MMBV’s bidirectional impact on prescribing, with reduced antibiotic use when the result indicated a viral etiology and increased, targeted prescribing when the result indicated a bacterial etiology, with fewer return hospitalizations (25). Collectively, these performance and utility data support MMBV as a tool to safely refine antibiotic and disposition decisions. However, real-world evidence is lacking.

Here, we conducted a pragmatic, real-world, pre-/post-implementation study to assess the utility of MMBV in children presenting to the ED with acute infections.

## 2 Methods

### 2.1 Ethics

Institutional Review Board (IRB) approval was obtained at Hillel Yaffe Medical Center (HYMC) (HYMC-0059-15, HYMC-0080-22), with waiver of consent under section 21 CFR 50.22. The study was conducted in accordance with the Declaration of Helsinki and applicable national and institutional standards.

### 2.2 Study design

This was a pragmatic, retrospective, single-center pre–post study. Medical records of pediatric patients presenting to the ED or pediatric inpatient unit of HYMC were assessed. “Pre” patients (constituting the standard-of-care arm, SC arm) were enrolled between 2014 and 2017 from a previously described pragmatic, retrospective cohort study (NCT03075111; “Spirit study”), in which MMBV was ordered at physician discretion, but results were not available in a timely or routine manner to inform practice (22). “Post” patients (constituting the MMBV arm) were enrolled between April 2021 and April 2024 in a pragmatic, retrospective cohort study, where MMBV was similarly ordered at physician discretion; however, in contrast to the pre-implementation study, testing was conducted in the laboratory during the patient encounter, so that results could influence routine clinical decisions. The MMBV arm was derived from an internal quality improvement evaluation of real-world MMBV implementation at HYMC. Because detailed clinical data extraction required manual chart review, full review of all tests cases was not feasible. The analytical cohort reflects a feasibility-based subset of tested cases that underwent chart abstraction and met the eligibility criteria for the present study. The transition from pre- to post-implementation included two critical activities: (1) switching from an ELISA-based measurement methodology to the rapid, 15-min MeMed Key® platform (MeMed, USA), enabling timely result availability for decision-making; and (2) physician retraining on intended use (including guidance that congenital or acquired immunodeficiency is a limitation on use), discussion of high-value use cases, and interpretation of MMBV results.

Antibiotic prescribing was defined as documented oral or intravenous antibiotic prescription given at any point up to discharge from the ED (for outpatients) or up to discharge from the hospital (for inpatients). The primary study objective was to compare antibiotic prescribing rates in the SC versus MMBV arms for outpatients with MMBV viral results. The primary endpoint was change in antibiotic prescribing. Secondary endpoints included the following:

(1) change in antibiotic prescribing between SC and MMBV arms stratified by disposition (inpatient vs. outpatient) and MMBV result (viral vs. bacterial);
(2) change in antibiotic prescribing for the subcohort of patients with LRTI, overall and stratified by disposition;
(3) change in hospital length of stay (LOS), overall and stratified by MMBV results; and
(4) association between antibiotic prescribing alignment with MMBV result and chest X-rays ordered, admissions, and LOS.

Discharge diagnoses were standardized using the Medical Dictionary for Regulatory Activities (MedDRA) and are listed in Supplementary Table S1. Diagnoses defining the LRTI subcohort of patients are listed in Supplementary Table S2.

### 2.3 Eligibility criteria

Children aged 3 months to 6 years (preschool age) were included if they had MMBV ordered at the physician’s discretion. For the SC arm, eligible patients were those recruited at HYMC in the previously described “Spirit” study. Across both arms, patients were excluded if they had missing MMBV, antibiotic prescription, or symptom duration data; if the medical record documented symptom duration exceeded 7 days; if fever was absent, or if antibiotic treatment had been initiated more than 48 h prior to presentation.

### 2.4 MeMed BV

MMBV (MeMed BV®, MeMed, US) integrates the levels of three host-immune proteins—TRAIL, CRP, and IP-10—to produce a score ranging from 0 to 100. Physicians were trained to clinically interpret scores according to the manufacturer’s instruction for use and validated thresholds (19–22, 26). A score below 35 indicates a viral or non-bacterial infection, a score between 35 and 65 is inconclusive (equivocal) but not invalid, a score above 65 but below 90 indicates a bacterial infection or co-infection, and a score above 90 strongly indicates a bacterial infection or co-infection.

For the SC arm, MMBV was performed using an ELISA-based platform (ImmunoXpert™, MeMed), with results available within 1–3 days. For the MMBV arm, testing was conducted on the rapid chemiluminescence-based platform (MeMed Key®) that delivered results within 15 min, enabling routine real-time use in clinical decision-making. Both platforms apply the same algorithm and yield comparable results (27).

### 2.5 Statistical analysis

Clinical and demographic data were summarized as descriptive statistics. Continuous variables were reported as either (i) median and interquartile range (IQR), with differences assessed using the Mann–Whitney U test or (ii) mean and standard deviation, with differences assessed using a *t*-test. Categorical variables were expressed as proportions, with differences assessed using Richardson’s method. Differences were considered statistically significant when *p*-value < 0.05. Statistical analysis was performed using Python version 3.9.4.

Comparisons were conducted between the SC and MMBV arms, and for subcohorts stratified by disposition (outpatients vs. inpatients) and by MMBV result. Additionally, within each study arm (SC and MMBV) analyzed separately, we compared clinical outcomes between patients whose antibiotic prescribing was aligned with their MMBV result and those for whom it was not. Alignment was defined as withholding antibiotics when the MMBV result was viral, and prescribing antibiotics when the result was bacterial. Patients with equivocal MMBV results were excluded from this analysis.

## 3 Results

### 3.1 Study cohort

**Figure 1:**
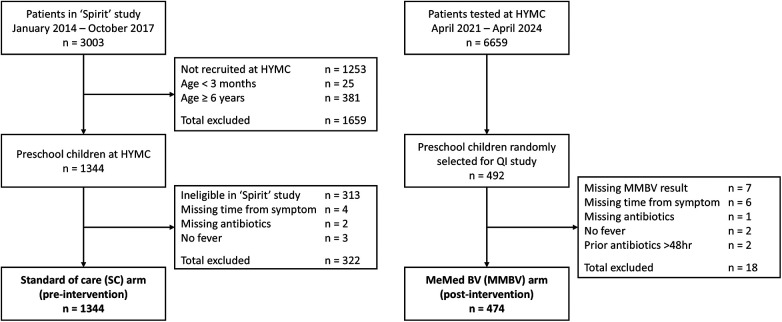
Study flow diagram for cohort derivation in the pre-implementation standard-of-care (SC) arm and post-implementation MeMed BV (MMBV) arm. In both arms, MMBV testing was ordered at physician discretion; however, results were available in real time only in the post-implementation MMBV arm. The transition from pre- to post-implementation included replacement of the ELISA-based platform with the 15-min MeMed Key® platform and physician retraining on intended use, high-value use cases, and interpretation of MMBV results.

The study cohort included 1,022 children in the SC arm and 474 in the MMBV arm (Figure 1). The proportion of girls (45.6% vs. 46.1%) and the median age [1.4 years (IQR: 0.8–2.5) vs. 1.3 years (IQR: 0.8–2.3)] were comparable across the SC and MMBV arms, with most children under 3 years of age (Supplementary Table S3). There were significant differences between the MMBV and SC arms in several characteristics, including prevalence of bacterial MMBV results (28.7% vs. 18.4%, *p* < 0.001), chest X-ray utilization (59.5% vs. 35.6%, *p* < 0.001), and rates of pneumonia (19.4% vs. 8.7%, *p* < 0.001). To address the imbalance across the SOC and MMBV arms, outpatients and inpatients were analyzed separately. Balanced patient demographics and clinical characteristics for outpatients and inpatients, pre- and post-implementation are provided in Table 1.

**Table 1:** Patient demographics, clinical characteristics, laboratory and microbiologic findings, and discharge diagnoses in the standard-of-care (SC; 2014–2017) and meMed BV (MMBV; 2021–2024) arms, stratified by outpatient vs. inpatient disposition.

	Outpatients			Inpatients		
	SC arm (2014–2017) (*n* = 541)	MMBV arm (2021–2024) (*n* = 107)	*P*-value*	SC arm (2014–2017) (*n* = 476)	MMBV arm (2021–2024) (*n* = 367)	*p*-value*
Sex, f; *n* (%)	238 (44.1%)	54 (50.5%)	0.243	225 (47.4%)	164 (44.8%)	0.486
Age, y; median (IQR)	1.3 (0.8, 2.5)	1.3 (0.8, 2.5)	0.783	1.5 (0.9, 2.5)	1.3 (0.8, 2.2)	0.141
3 m – 3y	432 (79.9%)	90 (84.1%)	0.351	369 (77.5%)	302 (82.3%)	0.101
3y – 6y	109 (20.1%)	17 (15.9%)	0.351	107 (22.5%)	65 (17.7%)	0.101
Time from symptoms onset, d; median (IQR)	3.0 (1.0, 4.0)	3.0 (1.0, 5.0)	0.389	2.0 (1.0, 4.0)	3.0 (1.0, 5.0)	<0.001
Temperature, °C; median (IQR)	39.5 (38.9, 40.0)	38.0 (37.3, 38.7)	0.0	39.6 (38.9, 40.0)	38.6 (37.9, 39.4)	<0.001
Main symptoms, *n* (%)						
Cough	217 (40.1%)	42 (39.3%)	0.914	191 (40.1%)	229 (62.4%)	<0.001
Dyspnea	33 (6.1%)	5 (4.7%)	0.821	73 (15.3%)	72 (19.6%)	0.118
Blood work						
CRP, mg/L; median (IQR)	20.6 (8.2, 42.3)	22.0 (6.8, 49.5)	0.806	37.2 (12.6, 90.4)	46.1 (16.1, 101.8)	0.181
WBC, ×10^9/L; median (IQR)	11.8 (8.6, 15.4)	11.5 (8.8, 15.6)	0.977	13.7 (9.8, 18.7)	14.1 (11.1, 19.5)	0.096
ANC, ×10^9/L; median (IQR)	6.1 (4.0, 9.0)	5.4 (3.5, 7.9)	0.187	8.0 (5.2, 12.6)	7.6 (5.1, 11.8)	0.547
MMBV; *n* (%)						
Bacterial	52 (9.6%)	11 (10.3%)	0.858	135 (28.4%)	125 (34.1%)	0.084
Equivocal	70 (12.9%)	16 (15.0%)	0.537	64 (13.4%)	46 (12.5%)	0.757
Viral	419 (77.4%)	80 (74.8%)	0.532	277 (58.2%)	196 (53.4%)	0.184
Chest x-ray; *n* (%)	122 (22.6%)	36 (33.6%)	0.019	241 (50.6%)	246 (67.0%)	<0.001
Microbiology; *n* (%)						
Adenovirus	0 (0.0%)	4 (3.7%)	0.001	17 (3.6%)	71 (19.3%)	<0.001
Influenza (A/B)	0 (0.0%)	5 (4.7%)	0.0	25 (5.3%)	14 (3.8%)	0.409
Rhino-/Enteroviruses	1 (0.2%)	2 (1.9%)	0.072	9 (1.9%)	43 (11.7%)	<0.001
RSV	1 (0.2%)	0 (0.0%)	1.0	15 (3.2%)	65 (17.7%)	<0.001
Admission rate, *n* (%)	0 (0.0%)	0 (0.0%)	None	476 (100.0%)	367 (100.0%)	None
Discharge diagnosis; *n* (%)						
LRTI	31 (5.7%)	7 (6.5%)	0.744	87 (18.3%)	152 (41.4%)	<0.001
URTI	123 (22.7%)	13 (12.1%)	0.014	79 (16.6%	48 (13.1%)	0.1571
Non-RTI	387 (71.5%)	87 (81.3%)	0.037	310 (65.1%)	167 (45.5%)	<0.001

IQR, Interquartile range; SD, standard deviation; CRP, C-reactive protein; WBC, white blood count; ANC, absolute neutrophil count; MMBV, MeMed BV; RTI, respiratory tract infection; RSV, respiratory syncytial virus; LRTI, lower respiratory tract infection; URTI, upper respiratory tract infection. *p*-values were calculated using the Mann–Whitney U test for ‘age’, ‘time from symptoms onset’, ‘length of stay’, ‘temperature’ and blood work variables. The remaining *p*-values were calculated using Richardson's method. Discharge diagnoses were coded using medDRA classification system; a full list is provided in Supplementary Table S1 and how they are grouped into LRTI and URTI is described in Supplementary Table S2.

As an exploratory analysis, we examined MMBV results among patients with detected respiratory viruses across the MMBV arm (Supplementary Table S4). Viral/non-bacterial MMBV results (<35) predominated in influenza-positive and RSV-positive cases and were also the most frequent in adenovirus-positive and rhino/enterovirus-positive cases. Notably, 19.8%–30.9% of cases with viral detection had bacterial MMBV results, possibly representing bacterial–viral co-infections. In patients with equivocal scores, virus detection by other microbiological testing was uncommon, occurring in only 18.3% of cases overall.

### 3.2 MMBV implementation and antibiotic prescribing

**Figure 2:**
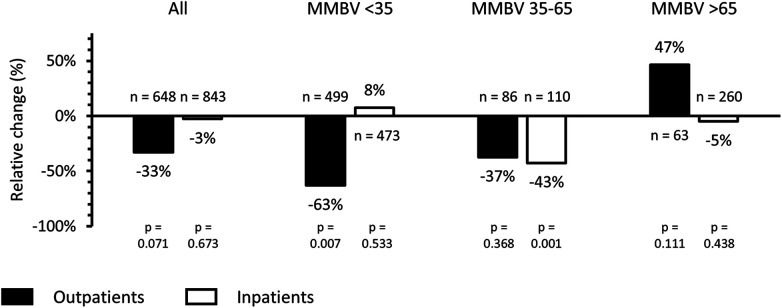
Relative percent change in antibiotic prescribing in the post-implementation MMBV arm compared with the pre-implementation SC arm, stratified by patient disposition and MMBV result category. Bars show the percent difference in antibiotic prescribing rates between arms for outpatients (black bars) and inpatients (white bars), stratified by MMBV result category: <35, viral/non-bacterial; 35–65, equivocal; and >65, bacterial/co-infection. Negative values indicate lower antibiotic prescribing in the MMBV arm than in the SC arm.

We compared antibiotic prescribing rates between the SC and MMBV arms, stratified by disposition (outpatients vs. inpatients) and MMBV results (Figure 2). There was a decrease in prescribing from 20.3% (*n* = 85/419) to 7.5% (*n* = 6/80; relative reduction 63%; *p* = 0.007) in the MMBV arm among outpatients with viral MMBV results (MMBV < 35), thereby achieving the primary endpoint. Among outpatients with bacterial MMBV results (MMBV > 65), antibiotic prescribing increased (*p* = 0.111). Overall outpatient prescribing decreased from 25.0% (*n* = 135/541) in the SC arm to 16.8% (*n* = 18/107) in the MMBV arm, a relative reduction of 33% (*p* = 0.071). This secondary endpoint did not meet statistical significance. There was no significant difference in overall inpatient antibiotic prescribing between arms (*p* = 0.673).

**Figure 3:**
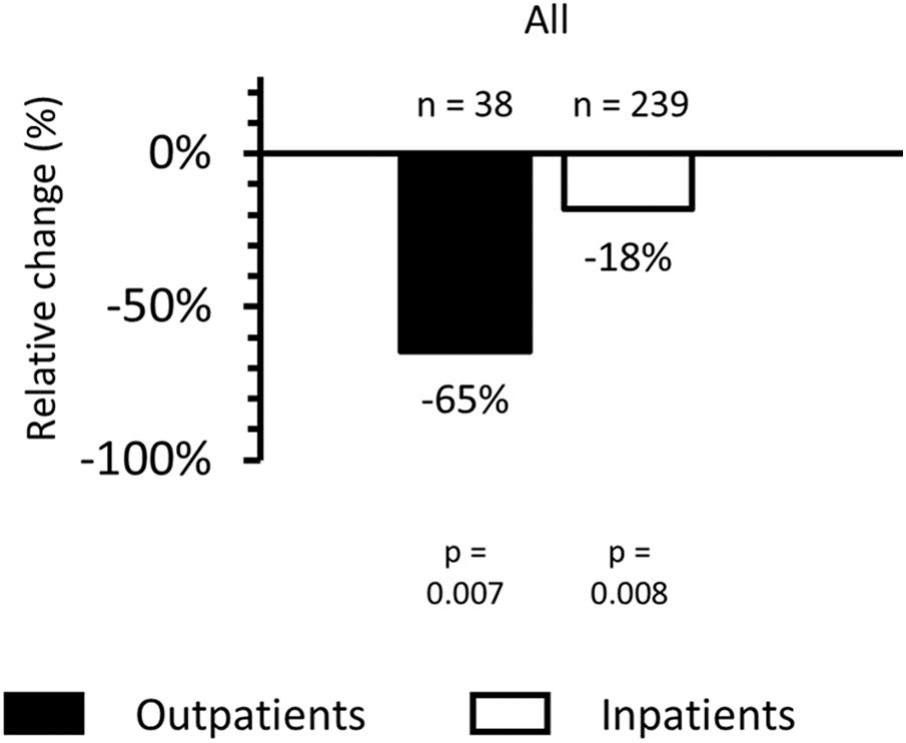
Relative percent change in antibiotic prescribing among patients with lower respiratory tract infection (LRTI) in the post-implementation MMBV arm compared with the pre-implementation SC arm, stratified by patient disposition. Bars show the percent difference in antibiotic prescribing rates between arms for outpatients (black bar) and inpatients (white bar). Negative values indicate lower antibiotic prescribing in the MMBV arm than in the SC arm.

Given the heightened diagnostic challenge in lower respiratory tract infections (LRTIs) due to confounding colonizers, co-infections, and sampling, we compared antibiotic prescribing rates in the SC and MMBV arms for patients discharged with LRTI (*n* = 278). Overall, prescribing decreased from 84.0% (*n* = 100/119) in the SC arm to 67.9% (*n* = 108/159 *p* = 0.002) in the MMBV arm. Prescribing reductions were observed both in outpatients [80.6% (*n* = 25/31) vs. 28.6% (*n* = 2/7); relative reduction 65%; *p* = 0.007] and inpatients [85.1% (*n* = 74/87) vs. 69.7% (*n* = 106/152); relative reduction 18%; *p* = 0.008; Figure 3].

### 3.3 MMBV implementation and hospital length of stay (LOS)

Despite more severe clinical presentation, patients in the MMBV arm had a shorter hospital length of stay (LOS) compared with those in the SC arm (Supplementary Table S3; 3.1 ± 1.9 vs. 3.6 ± 1.9 days; *p* < 0.001). This shorter LOS was observed in patients with viral MMBV results (3.0 ± 1.9 vs. 3.4 ± 1.7 days; *p* = 0.033) and bacterial MMBV results (3.2 ± 1.7 vs. 4.0 ± 2.5 days; *p* = 0.005), but not in those with equivocal MMBV results (*p* = 0.725).

### 3.4 Association between alignment of antibiotic prescribing with MMBV result and chest X-rays ordered, admissions, and LOS

To further explore clinical benefit, we compared outcomes between patients whose antibiotic management aligned with the MMBV result and those whose antibiotic management did not. We hypothesized that any differences would be more pronounced in the SC arm, where MMBV was not used to guide prescribing.

In the SC arm, among patients with viral MMBV results, alignment (i.e., not prescribing antibiotics) was associated with fewer admissions (34.5% vs. 54.3%; *p* < 0.001), shorter LOS (3.0 ± 1.2 vs. 4.1 ± 2.1 days; *p* < 0.001), and fewer chest X-rays ordered (19.3% vs. 47.8%; *p* < 0.001; Supplementary Table S5). Conversely, among patients with bacterial MMBV results, alignment (i.e., prescribing antibiotics) was associated with higher admission rates (79.0% vs. 53.1%; *p* < 0.001) and longer LOS (4.3 ± 2.6 vs. 2.7 ± 1.4 days; *p* < .001), but not with chest X-rays ordered (68.3% vs. 53.1%; *p* = 0.059; Supplementary Table S6).

In the MMBV arm, the associations between prescribing alignment and patient outcomes were attenuated. Among patients with viral MMBV results, the LOS difference was no longer significant (2.9 ± 2.0 vs. 3.2 ± 1.7 days; *p* = 0.226) (Supplementary Table S7). No significant associations were observed between prescribing alignment and outcomes among patients with bacterial MMBV results (Supplementary Table S8).

## 4 Discussion

This real-world study supports that MMBV implementation influences clinical decision-making for pediatric patients presenting with acute infections. We observed significantly lower antibiotic prescribing in pediatric outpatients with viral MMBV scores after implementation. For patients with LRTI, reductions in prescription were observed, irrespective of patient disposition. In addition, we observed a reduction in the hospital LOS upon implementation of MMBV. Lastly, we found that alignment of antibiotic prescribing with viral MMBV results (i.e., no prescription) was associated with fewer admissions, shorter LOS, and fewer chest X-rays ordered. In an exploratory analysis, we identified that a significant proportion of patients with detected respiratory viruses had non-viral MMBV results, highlighting that microbiologic detection does not necessarily establish causation.

Antimicrobial stewardship aims to reduce unwarranted antibiotic exposure to curb the development of antimicrobial resistance and prevent avoidable adverse events. Reducing unwarranted antibiotic use must be balanced against the risk of undertreating patients who would benefit from antibiotics, such as those with potentially serious bacterial infections. Consequently, novel diagnostic tools designed to rapidly differentiate bacterial from viral infection etiologies must demonstrate high sensitivity and specificity. Importantly, when assessing the utility of such tools, a bidirectional change in practice should be expected, depending on the test result. Our findings support that MMBV contributes by significantly reducing antibiotic prescriptions in patients with viral MMBV scores, while directing antibiotic use in patients with bacterial MMBV scores. These opposing effects explain the small net reduction in antibiotic use for outpatients and, consequently, why this secondary endpoint was not achieved. Unlike pathogen detection tests, such as cultures, which yield negative results in as many as 90% of cases, MMBV yields equivocal results in approximately 10%–13% of cases. As these results are non-actionable (according to manufacturer specifications), they may be considered a source of frustration. Notably, we observed significant reductions in antibiotic prescribing among both outpatients and inpatients with equivocal results, consistent with findings from randomized adult trials. This suggests that equivocal results may influence physician decision-making in practice, likely reflecting pre-MMBV test suspicion for bacterial infection.

The results of this study align with post-COVID real-world evidence demonstrating that MMBV influences and optimizes physician treatment decisions (28, 29) and is associated with fewer hospital admissions (28, 30). Indeed, beyond optimized antibiotic prescribing, randomized controlled trials in adults (25) have shown that MMBV-guided treatment reduces return hospitalizations, while pediatric case–control data (24) have linked MMBV availability to reduced multiplex PCR testing. Taken together with the present findings, accumulating utility data support MMBV as a tool that optimizes antibiotic prescribing, leading to better patient outcomes and optimized healthcare utilization.

Several factors likely influenced the modest impact of MMBV on antibiotic prescribing that we observed for inpatients. The timing of testing was most notable; if MMBV was ordered after admission, antibiotics may already have been initiated in the ED. Accordingly, even if the antibiotics were discontinued in the ward, as the study endpoint was antibiotic use at any point up to hospital discharge, MMBV’s impact would not have been detected. We suspect that this issue may have contributed to the undetectable impact of MMBV on antibiotic prescribing for inpatients in the present study. Future utility studies should focus on inpatients, incorporate training of carers throughout the patient journey, and assess the timing and type of antibiotic prescribing.

We observed a significant reduction in LOS in the MMBV arm. While this reduction may reflect a broader healthcare trend toward shorter patient LOS, it is notable that a significant proportion of inpatients had an LRTI (278/1496, 18.6%), for whom LOS has reportedly remained stable over the past decade (31). Moreover, we observed significant reductions in LOS in patients with viral or bacterial MMBV results but not in those with equivocal results. This pattern suggests that MMBV-guided antimicrobial prescribing may reduce clinical uncertainty and contribute to shorter hospital stays. Further support for this interpretation is provided by the correlative finding that, when we aligned antibiotic prescribing practice with MMBV results, we observed lower admission rates, shorter LOS, and fewer chest X-rays ordered among patients with viral MMBV results in the SC arm. Taken together, these data support an operational and potentially cost-effective benefit for hospitals implementing MMBV. More generally, the study’s findings align with recent cost-effectiveness analyses, showing that improved antibiotic stewardship and more accurate disposition decisions enabled by MMBV can yield substantial cost savings for hospitals (24, 32, 33). Looking ahead, randomized controlled trials in children with suspected LRTI are warranted to directly evaluate whether MMBV safely impacts antibiotic prescribing, clinical outcomes, and cost savings for both outpatients and inpatients.

This study has several limitations. The study was retrospective in design and therefore constrained by the data available and sample size; for example, information on ED duration, pneumonia severity, and follow-up visits was not available and cost impact assessment was out of scope. Another limitation is that the post-implementation cohort was derived from a feasibility-based subset of all MMBV tests performed during the study period. Consequently, selection bias may have contributed to the baseline differences observed between the SC and MMBV arms. Nonetheless, the apparent higher acuity among patients in the MMBV arm may also reflect real-world ordering patterns, with preferential use in children under consideration for admission, for whom blood was drawn due to uncertainty about bacterial infection. Stratified analyses were conducted of outpatients and inpatients to help mitigate this bias. An additional limitation is that the intervention phase overlapped with the COVD-19 pandemic and the post-pandemic recovery period, during which infection control practices, testing pathways, and hospital workflows differed from the pre-pandemic control period (16, 31, 34, 35). These changes may also have contributed to baseline differences between the study arms, including higher respiratory burden and apparent acuity of patients in the MMBV arm. Notably, however, such pandemic-induced pressures would be expected to bias outcomes toward greater clinical caution rather than toward improved stewardship. In this context, the shorter LOS observed in the MMBV arm despite more severe clinical presentation, together with the bidirectional changes in antibiotic prescribing according MMBV result categories, suggests that the observed effects were at least partly attributable to MMBV implementation rather than solely to temporal confounding. Finally, the single-center design limits the generalizability of our findings. Future multi-center implementation studies are needed to establish use cases where MMBV provides the highest value.

A key strength of this study is its pragmatic design, which provides insights into the real-world implementation of MMBV testing. Importantly, MMBV was performed for patients in the SC arm, which enabled patient stratification, revealing the test’s bidirectional impact on antibiotic prescribing.

## 5 Conclusions

Our study supports that MMBV implementation guides antibiotic use and is associated with reduced hospital length of stay. These findings indicate that MMBV supports appropriate antimicrobial prescribing and is associated with improved clinical outcomes.

## Data Availability

The raw data supporting the conclusions of this article will be made available by the authors, without undue reservation, upon reasonable request to the corresponding author with a clearly stated purpose.
