# Correction: Clinical utility of a host-protein test for suspected infection in the pediatric emergency department: a pragmatic pre-/post-implementation study

**DOI:** 10.3389/fped.2026.1886703

**Published:** 2026-06-16

**Authors:** Vered Nir, Vered Schichter Konfino, Naama Kuchinski Cohen, Esther Levy, Noa Kremer, Yosef Or Shamia, Amir Nakar, Boris Lebedenko, Jeroen Stas, Tanya M. Gottlieb, Ma’anit Shapira, Michal Stein, Adi Klein

**Affiliations:** 1 Pediatrics Department, Hillel Yaffe Medical Center, Hadera, Israel; 2 Rappaport Faculty of Medicine, Technion Institute of Technology, Haifa, Israel; 3 Pediatrics Emergency Department, Hillel Yaffe Medical Center, Hadera, Israel; 4 Clinical Laboratory Division, Hillel Yaffe Medical Center, Hadera, Israel; 5 Pediatrics Department, Carmel Medical Center, Haifa, Israel; 6 MeMed, Tirat Carmel, Israel; 7 Pediatric Infectious Diseases Unit, Sheba Medical Center, Edmond and Lily Safra Children’s Hospital, Tel- Hashomer, Israel; 8 Gray Faculty of Medicine, Tel Aviv University, Tel Aviv, Israel

**Keywords:** antibiotic stewardship, biomarkers, emergency department, host-response testing, MeMed BV

## Error in figure/table

Wrong content

There was a mistake in Table 1 as published. In Table 1, there were numbers provided on the line “MMBV; *n* (%) for the inpatient columns but not for the outpatient columns on the same line. No information/numbers should have been listed on this line. Similarly, there was a blank line below the line “RSV” with only numbers for the inpatient columns. This information for the inpatient columns should have been provided on the line below called “admission rate, *n* (%)” where currently the wrong numbers are displayed. The corrected Table 1 appears below.

**Table 1:** Patient demographics, clinical characteristics, laboratory and microbiologic findings, and discharge diagnoses in the standard-of-care (SC; 2014–2017) and meMed BV (MMBV; 2021–2024) arms, stratified by outpatient vs. inpatient disposition.

	Outpatients			Inpatients		
	SC arm (2014–2017) (*n* = 541)	MMBV arm (2021–2024) (*n* = 107)	*P*-value*	SC arm (2014–2017) (*n* = 476)	MMBV arm (2021–2024) (*n* = 367)	*p*-value*
Sex, f; *n* (%)	238 (44.1%)	54 (50.5%)	0.243	225 (47.4%)	164 (44.8%)	0.486
Age, y; median (IQR)	1.3 (0.8, 2.5)	1.3 (0.8, 2.5)	0.783	1.5 (0.9, 2.5)	1.3 (0.8, 2.2)	0.141
3 m – 3y	432 (79.9%)	90 (84.1%)	0.351	369 (77.5%)	302 (82.3%)	0.101
3y – 6y	109 (20.1%)	17 (15.9%)	0.351	107 (22.5%)	65 (17.7%)	0.101
Time from symptoms onset, d; median (IQR)	3.0 (1.0, 4.0)	3.0 (1.0, 5.0)	0.389	2.0 (1.0, 4.0)	3.0 (1.0, 5.0)	<0.001
Temperature, °C; median (IQR)	39.5 (38.9, 40.0)	38.0 (37.3, 38.7)	0.0	39.6 (38.9, 40.0)	38.6 (37.9, 39.4)	<0.001
Main symptoms, *n* (%)						
Cough	217 (40.1%)	42 (39.3%)	0.914	191 (40.1%)	229 (62.4%)	<0.001
Dyspnea	33 (6.1%)	5 (4.7%)	0.821	73 (15.3%)	72 (19.6%)	0.118
Blood work						
CRP, mg/L; median (IQR)	20.6 (8.2, 42.3)	22.0 (6.8, 49.5)	0.806	37.2 (12.6, 90.4)	46.1 (16.1, 101.8)	0.181
WBC, ×10^9/L; median (IQR)	11.8 (8.6, 15.4)	11.5 (8.8, 15.6)	0.977	13.7 (9.8, 18.7)	14.1 (11.1, 19.5)	0.096
ANC, ×10^9/L; median (IQR)	6.1 (4.0, 9.0)	5.4 (3.5, 7.9)	0.187	8.0 (5.2, 12.6)	7.6 (5.1, 11.8)	0.547
MMBV; *n* (%)						
Bacterial	52 (9.6%)	11 (10.3%)	0.858	135 (28.4%)	125 (34.1%)	0.084
Equivocal	70 (12.9%)	16 (15.0%)	0.537	64 (13.4%)	46 (12.5%)	0.757
Viral	419 (77.4%)	80 (74.8%)	0.532	277 (58.2%)	196 (53.4%)	0.184
Chest x-ray; *n* (%)	122 (22.6%)	36 (33.6%)	0.019	241 (50.6%)	246 (67.0%)	<0.001
Microbiology; *n* (%)						
Adenovirus	0 (0.0%)	4 (3.7%)	0.001	17 (3.6%)	71 (19.3%)	<0.001
Influenza (A/B)	0 (0.0%)	5 (4.7%)	0.0	25 (5.3%)	14 (3.8%)	0.409
Rhino-/Enteroviruses	1 (0.2%)	2 (1.9%)	0.072	9 (1.9%)	43 (11.7%)	<0.001
RSV	1 (0.2%)	0 (0.0%)	1.0	15 (3.2%)	65 (17.7%)	<0.001
Admission rate, *n* (%)	0 (0.0%)	0 (0.0%)	None	476 (100.0%)	367 (100.0%)	None
Discharge diagnosis; *n* (%)						
LRTI	31 (5.7%)	7 (6.5%)	0.744	87 (18.3%)	152 (41.4%)	<0.001
URTI	123 (22.7%)	13 (12.1%)	0.014	79 (16.6%	48 (13.1%)	0.1571
Non-RTI	387 (71.5%)	87 (81.3%)	0.037	310 (65.1%)	167 (45.5%)	<0.001

IQR, Interquartile range; SD, standard deviation; CRP, C-reactive protein; WBC, white blood count; ANC, absolute neutrophil count; MMBV, MeMed BV; RTI, respiratory tract infection; RSV, respiratory syncytial virus; LRTI, lower respiratory tract infection; URTI, upper respiratory tract infection. *p*-values were calculated using the Mann–Whitney U test for ‘age’, ‘time from symptoms onset’, ‘length of stay’, ‘temperature’ and blood work variables. The remaining *p*-values were calculated using Richardson's method. Discharge diagnoses were coded using medDRA classification system; a full list is provided in Supplementary Table S1 and how they are grouped into LRTI and URTI is described in Supplementary Table S2.

The original article has been updated.

