# Supplementary material for: Clinical utility of a host-protein test for suspected infection in the pediatric emergency department: a pragmatic pre-/post-implementation study
Source: Front Pediatr. 2026 May 15;14:1803886. doi: 10.3389/fped.2026.1803886 (PMC13219261; doi:10.3389/fped.2026.1803886)
Supplement: Supplementary file 1 [file Table1.docx]

Supplementary Material

# Supplementary tables

**Table S1**: List of discharge diagnosis frequency per MedDRA dictionary classification.

| **Discharge Diagnosis per MedDRA classification** | **n*** | **(%)** |
| --- | --- | --- |
| Abscess | 7 | (0.5) |
| Appendicitis | 6 | (0.4) |
| Arthritis bacterial | 1 | (0.1) |
| Arthritis reactive | 1 | (0.1) |
| Arthropathy | 1 | (0.1) |
| Asthma | 2 | (0.1) |
| Bacteraemia | 17 | (1.1) |
| Bacterial infection | 1 | (0.1) |
| Bronchiolitis | 102 | (6.8) |
| Bronchitis | 1 | (0.1) |
| Cellulitis | 5 | (0.3) |
| Conjunctivitis | 4 | (0.3) |
| Diarrhoea infectious | 1 | (0.1) |
| Dyspnoea | 1 | (0.1) |
| Familial mediterranean fever | 1 | (0.1) |
| Febrile convulsion | 43 | (2.9) |
| Gastroenteritis | 38 | (2.5) |
| H1N1 influenza | 1 | (0.1) |
| Hand-foot-and-mouth disease | 2 | (0.1) |
| Herpangina | 10 | (0.7) |
| Ill-defined disorder | 43 | (2.9) |
| Immunisation reaction | 1 | (0.1) |
| Infectious mononucleosis | 6 | (0.4) |
| Influenza | 5 | (0.3) |
| Kawasaki's disease | 1 | (0.1) |
| Laryngitis | 2 | (0.1) |
| Lower respiratory tract infection | 16 | (1.1) |
| Lymphadenitis | 3 | (0.2) |
| Mastoiditis | 5 | (0.3) |
| Meningitis | 17 | (1.1) |
| Multisystem inflammatory syndrome in children | 1 | (0.1) |
| Myringitis | 1 | (0.1) |
| Oral herpes | 5 | (0.3) |
| Osteomyelitis | 3 | (0.2) |
| Otitis externa | 2 | (0.1) |
| Otitis media acute | 79 | (5.3) |
| Parotitis | 1 | (0.1) |
| Periorbital abscess | 3 | (0.2) |
| Periorbital cellulitis | 2 | (0.1) |
| Pharyngitis | 4 | (0.3) |
| Pneumonia | 181 | (12.1) |
| Pyelonephritis | 25 | (1.7) |
| Pyrexia | 80 | (5.4) |
| Scarlet fever | 1 | (0.1) |
| Synovitis | 3 | (0.2) |
| Tonsillitis | 75 | (5.0) |
| Upper respiratory tract infection | 123 | (8.2) |
| Urinary tract infection | 27 | (1.8) |
| Viral infection | 541 | (36.2) |
| Wheezing | 1 | (0.1) |

With MedDRA, Medical Dictionary for Regulatory Activities.

* Some patients had multiple discharge diagnoses noted in their medical records.

**Table S2:** MedDRA discharge diagnoses grouped under the labels URTI and LRTI.

| **Lower Respiratory Tract Infections (LRTI)** |
| --- |
| Asthma |
| Bronchiolitis |
| Bronchitis |
| H1N1 Influenza |
| Influenza |
| Lower Respiratory Tract Infection |
| Pneumonia |
| **Upper Respiratory Tract Infections (URTI)** |
| Conjunctivitis |
| Herpangina |
| Laryngitis |
| Mastoiditis |
| Myringitis |
| Otitis externa |
| Otitis media acute |
| Pharyngitis |
| Tonsilitis |
| Upper respiratory tract infection |

**Table S3.** Patient demographics and clinical characteristics.

|  | **SC**  **(2014-2017)**  (n = 1022) | **MMBV**  **(2021-2024)**  (n = 474) | **p-value** |
| --- | --- | --- | --- |
| Sex, f; n (%) | 465 (45.6%) | 218 (46.1%) | 0.857 |
| Age, y; median (IQR) | 1.4 (0.8, 2.5) | 1.3 (0.8, 2.3) | 0.301 |
| 3m – 3y | 806 (78.9%) | 392 (82.7%) | 0.0841 |
| 3y – 6y | 216 (21.1%) | 82 (17.3%) | 0.0841 |
| Time from symptoms onset, median (IQR) | 2.0 (1.0, 4.0) | 3.0 (1.0, 5.0) | <0.001 |
| Temperature, °C; median (IQR) | 39.5 (38.9, 40.0) | 38.5 (37.7, 39.2) | <0.001 |
| Main symptoms, n (%) |  |  |  |
| Cough | 410 (40.1%) | 271 (57.2%) | <0.001 |
| Dyspnea | 106 (10.4%) | 77 (16.2%) | 0.001 |
| Blood work |  |  |  |
| CRP, mg/l; median (IQR) | 25.8 (9.7, 58.4) | 37.0 (12.0, 79.3) | <0.001 |
| WBC, x 10^9/l; median (IQR) | 12.5 (9.1, 17.1) | 13.6 (10.4, 18.5) | <0.001 |
| ANC, x 10^9/l; median (IQR) | 6.8 (4.4, 10.4) | 7.0 (4.8, 11.0) | 0.157 |
| MMBV; n (%) |  |  |  |
| Viral | 699 (68.4%) | 276 (58.2%) | <0.001 |
| Equivocal | 135 (13.2%) | 62 (13.1%) | 0.945 |
| Bacterial | 188 (18.4%) | 136 (28.7%) | <0.001 |
| Chest x-ray; n (%) | 364 (35.6%) | 282 (59.5%) | <0.001 |
| Microbiology; n (%) |  |  |  |
| Adenovirus | 17 (1.7%) | 75 (15.8%) | <0.001 |
| Influenza (A/B) | 26 (2.5%) | 19 (4.0%) | 0.123 |
| Rhino-/Enteroviruses | 10 (1.0%) | 45 (9.5%) | <0.001 |
| RSV | 16 (1.6%) | 65 (13.7%) | <0.001 |
| Admission rate, n (%) | 476 (46.6%) | 367 (77.4%) | <0.001 |
| Length of stay, d; mean (SD) | 3.6 (1.9%) | 3.1 (1.9%) | <0.001 |
| Discharge diagnosis group; n (%) |  |  |  |
| LRTI | 119 (11.6%) | 159 (33.5%) | <0.001 |
| URTI | 204 (20.0%) | 61 (12.9%) | 0.0008 |
| Non-RTI | 699 (68.4%) | 254 (53.6%) | <0.001 |

IQR, Interquartile range; SD, standard deviation; CRP, C-reactive protein; WBC, white blood count; ANC, absolute neutrophil count; MMBV, MeMed BV; RTI, respiratory tract infection; RSV, respiratory syncytial virus; LRTI, lower respiratory tract infection; URTI, upper respiratory tract infection.

p-values were calculated using the Mann-Whitney U test for ‘age’, ‘time from symptoms onset’, ‘length of stay’, ‘temperature’ and blood work variables. The remaining p-values were calculated using Richardson's method.

Discharge diagnoses were coded using medDRA classification system; a full list is provided in supplementary table 1 and how they are grouped into LRTI and URTI is described in supplementary table 2.

**Table S4.** MMBV score distribution among detected respiratory virus cases in the MMBV arm.

|  | **Influenza**  **(n = 45)** | **RSV**  **(n = 81)** | **Adenovirus**  **(n = 92)** | **Rhino-/Enteroviruses**  **(n = 55)** |
| --- | --- | --- | --- | --- |
| MMBV <35; n (%) | 34 (75.6%) | 53 (65.4%) | 49 (53.3%) | 34 (61.8%) |
| MMBV 35-65; n (%) | 2 (4.4%) | 12 (14.8%) | 15 (16.3%) | 4 (7.3%) |
| MMBV >65; n (%) | 9 (20.0%) | 16 (19.8%) | 28 (30.4%) | 17 (30.9%) |
| MMBV score: median (Q1-Q3) | 8.0 (0.0-31.0) | 16.0 (4.0-61.0) | 27.5 (5.8-79.2) | 18.0 (3.0-83.0) |

MMBV, MeMed BV; RSV, respiratory syncytial virus

**Table S5.** SC arm patient demographics with MMBV <35, by antibiotic prescription alignment.

|  | **MMBV <35 & no Abx**  **(n = 513)** | **MMBV <35 & Abx**  **(n = 186)** | **p-value*** |
| --- | --- | --- | --- |
| Sex, f; n (%) | 213 (41.6%) | 96 (51.9%) | 0.016 |
| Age, y; median (IQR) | 1.2 (0.8, 2.2) | 1.3 (0.8, 2.0) | 0.323 |
| 3m – 3y | 435 (84.8%) | 156 (83.9%) | 0.765 |
| 3y – 6y | 78 (15.2%) | 30 (16.1%) | 0.765 |
| Time from symptoms onset, median (IQR) | 2.0 (1.0, 4.0) | 2.0 (1.0, 4.0) | 0.617 |
| Temperature, °C; median (IQR) | 39.5 (38.9, 40.0) | 39.5 (38.8, 40.0) | 0.351 |
| Main symptoms, n (%) |  |  |  |
| Cough | 199 (38.8%) | 93 (50.0%) | 0.008 |
| Dyspnea | 39 (7.6%) | 34 (18.3%) | <0.001 |
| Blood work |  |  |  |
| CRP, mg/l; median (IQR) | 14.1 (5.7, 28.1) | 21.9 (10.8, 42.6) | <0.001 |
| WBC, x 10^9/l; median (IQR) | 11.2 (8.3, 14.9) | 12.4 (9.8, 16.7) | 0.002 |
| ANC, x 10^9/l; median (IQR) | 5.6 (3.8, 8.4) | 7.0 (4.4, 9.5) | <0.001 |
| Chest x-ray; n (%) | 99 (19.3%) | 89 (47.8%) | <0.001 |
| Microbiology; n (%) | 3 (0.6%) | 4 (2.2%) | 0.066 |
| Adenovirus | 13 (2.5%) | 9 (4.8%) | 0.123 |
| Influenza (A/B) | 2 (0.4%) | 4 (2.2%) | 0.026 |
| Rhino-/Enteroviruses | 5 (1.0%) | 6 (3.2%) | 0.035 |
| RSV | 19 (3.7%) | 49 (26.3%) | <0.001 |
| Admission rate, n (%) | 176 (34.5%) | 101 (54.3%) | <0.001 |
| Length of stay, d; mean (SD) | 3.0 (1.2) | 4.1 (2.1) | <0.001 |
| Discharge diagnosis group; n (%) | 401 (78.2%) | 88 (47.3%) | <0.001 |
| LRTI | 93 (18.1%) | 49 (26.3%) | 0.017 |
| URTI | 99 (19.3%) | 89 (47.8%) | <0.001 |
| Non-RTI | 3 (0.6%) | 4 (2.2%) | 0.066 |

IQR, Interquartile range; SD, standard deviation; CRP, C-reactive protein; WBC, white blood count; ANC, absolute neutrophil count; MMBV, MeMed BV; RTI, respiratory tract infection; RSV, respiratory syncytial virus; LRTI, lower respiratory tract infection; URTI, upper respiratory tract infection.

p-values were calculated using the Mann-Whitney U test for ‘age’, ‘time from symptoms onset’, ‘length of stay’, ‘temperature’ and blood work variables. The remaining p-values were calculated using Richardson's method.

Discharge diagnoses were coded using medDRA classification system; a full list is provided in supplementary table 1 and how they are grouped into LRTI and URTI is described in supplementary table 2.

**Table S6:** SC arm patient demographics with MMBV >65, by antibiotic prescription alignment.

|  | **MMBV >65 & no Abx**  **(n = 49)** | **MMBV >65 & Abx**  **(n = 139)** | **p-value*** |
| --- | --- | --- | --- |
| Sex, f; n (%) | 20 (40.8%) | 75 (54.0%) | 0.115 |
| Age, y; median (IQR) | 2.0 (1.2, 3.0) | 1.7 (1.2, 3.5) | 0.988 |
| 3m – 3y | 33 (67.3%) | 91 (65.5%) | 0.812 |
| 3y – 6y | 16 (32.7%) | 48 (34.5%) | 0.812 |
| Time from symptoms onset, median (IQR) | 3.0 (1.0, 4.0) | 3.0 (1.0, 4.0) | 0.483 |
| Temperature, °C; median (IQR) | 39.7 (38.8, 40.1) | 39.8 (39.0, 40.1) | 0.261 |
| Main symptoms, n (%) |  |  |  |
| Cough | 16 (32.7%) | 54 (38.8%) | 0.442 |
| Dyspnea | 2 (4.1%) | 16 (11.5%) | 0.130 |
| Blood work |  |  |  |
| CRP, mg/l; median (IQR) | 69.0 (41.3, 128.2) | 121.9 (79.3, 175.4) | <0.001 |
| WBC, x 10^9/l; median (IQR) | 12.5 (9.3, 17.1) | 17.1 (12.9, 22.0) | <0.001 |
| ANC, x 10^9/l; median (IQR) | 7.7 (5.0, 11.5) | 11.5 (7.8, 16.5) | <0.001 |
| Chest x-ray; n (%) | 26 (53.1%) | 95 (68.3%) | 0.055 |
| Microbiology; n (%) |  |  |  |
| Adenovirus | 0 (0.0%) | 4 (2.9%) | 0.231 |
| Influenza (A/B) | 0 (0.0%) | 4 (2.9%) | 0.231 |
| Rhino-/Enteroviruses | 0 (0.0%) | 3 (2.2%) | 0.301 |
| RSV | 0 (0.0%) | 4 (2.9%) | 0.231 |
| Admission rate, n (%) | 26 (53.1%) | 109 (79.0%) | <0.001 |
| Length of stay, d; mean (SD) | 2.7 (1.4) | 4.3 (2.6) | 0.003 |
| Discharge diagnosis group; n (%) |  |  |  |
| LRTI | 0 (0.0%) | 35 (25.2%) | <0.001 |
| URTI | 35 (71.4%) | 90 (64.7%) | 0.396 |
| Non-RTI | 14 (28.6%) | 14 (10.1%) | 0.002 |

IQR, Interquartile range; SD, standard deviation; CRP, C-reactive protein; WBC, white blood count; ANC, absolute neutrophil count; MMBV, MeMed BV; RTI, respiratory tract infection; RSV, respiratory syncytial virus; LRTI, lower respiratory tract infection; URTI, upper respiratory tract infection.

p-values were calculated using the Mann-Whitney U test for ‘age’, ‘time from symptoms onset’, ‘length of stay’, ‘temperature’ and blood work variables. The remaining p-values were calculated using Richardson's method.

Discharge diagnoses were coded using medDRA classification system; a full list is provided in supplementary table 1 and how they are grouped into LRTI and URTI is described in supplementary table 2.

**Table S7:** MMBV arm patient demographics with MMBV <35, by antibiotic prescription alignment.

|  | **MMBV <35 & no Abx**  **(n = 193)** | **MMBV <35 & Abx**  **(n = 83)** | **p-value** |
| --- | --- | --- | --- |
| Sex, f; n (%) | 86 (44.6%) | 36 (43.4%) | 0.856 |
| Age, y; median (IQR) | 1.2 (0.7, 2.0) | 1.3 (0.9, 1.9) | 0.578 |
| 3m – 3y | 169 (87.6%) | 72 (86.7%) | 0.852 |
| 3y – 6y | 24 (12.4%) | 11 (13.3%) | 0.852 |
| Time from symptoms onset, median (IQR) | 3.0 (1.0, 5.0) | 3.0 (2.0, 4.0) | 0.981 |
| Temperature, °C; median (IQR) | 38.3 (37.6, 39.0) | 38.4 (37.7, 39.2) | 0.640 |
| Main symptoms, n (%) |  |  |  |
| Cough | 104 (53.9%) | 52 (62.7%) | 0.179 |
| Dyspnea | 26 (13.5%) | 21 (25.3%) | 0.017 |
| Blood work |  |  |  |
| CRP, mg/l; median (IQR) | 15.0 (5.0, 36.0) | 28.0 (11.5, 62.0) | <0.001 |
| WBC, x 10^9/l; median (IQR) | 12.2 (9.7, 15.6) | 13.2 (9.9, 16.9) | 0.339 |
| ANC, x 10^9/l; median (IQR) | 5.7 (4.2, 8.9) | 7.4 (3.8, 10.5) | 0.108 |
| Chest x-ray; n (%) | 78 (40.4%) | 59 (71.1%) | <0.001 |
| Microbiology; n (%) |  |  |  |
| Adenovirus | 32 (16.6%) | 10 (12.0%) | 0.337 |
| Influenza (A/B) | 9 (4.7%) | 3 (3.6%) | 0.696 |
| Rhino-/Enteroviruses | 17 (8.8%) | 11 (13.3%) | 0.263 |
| RSV | 22 (11.4%) | 20 (24.1%) | 0.007 |
| Admission rate, n (%) | 119 (61.7%) | 77 (92.8%) | <0.001 |
| Length of stay, d; mean (SD) | 2.9 (2.0) | 3.2 (1.7) | 0.240 |
| Discharge diagnosis group; n (%) |  |  |  |
| LRTI | 40 (20.7%) | 43 (51.8%) | <0.001 |
| URTI | 136 (70.5%) | 28 (33.7%) | <0.001 |
| Non-RTI | 17 (8.8%) | 12 (14.5%) | 0.161 |

IQR, Interquartile range; SD, standard deviation; CRP, C-reactive protein; WBC, white blood count; ANC, absolute ne-trophil count; MMBV, MeMed BV; RTI, respiratory tract infection; RSV, respiratory syncytial virus; LRTI, lower respiratory tract infection; URTI, upper respiratory tract infection.

p-values were calculated using the Mann-Whitney U test for ‘age’, ‘time from symptoms onset’, ‘length of stay’, ‘temperature’ and blood work variables. The remaining p-values were calculated using Richardson's method.

Discharge diagnoses were coded using medDRA classification system; a full list is provided in supplementary table 1 and how they are grouped into LRTI and URTI is described in supplementary table 2.

**Table S8:** MMBV arm patient demographics with MMBV >65, by antibiotic prescription alignment.

|  | **MMBV >65 & no Abx**  **(n = 31)** | **MMBV >65 & Abx**  **(n = 105)** | **p-value** |
| --- | --- | --- | --- |
| Sex, f; n (%) | 13 (41.9%) | 50 (48.1%) | 0.549 |
| Age, y; median (IQR) | 2.1 (1.1, 2.9) | 1.5 (0.9, 2.5) | 0.260 |
| 3m – 3y | 24 (77.4%) | 82 (78.1%) | 0.937 |
| 3y – 6y | 7 (22.6%) | 23 (21.9%) | 0.937 |
| Time from symptoms onset, median (IQR) | 3.0 (1.5, 5.0) | 3.0 (2.0, 4.0) | 0.768 |
| Temperature, °C; median (IQR) | 39.0 (37.8, 39.7) | 38.8 (37.8, 39.5) | 0.605 |
| Main symptoms, n (%) |  |  |  |
| Cough | 20 (64.5%) | 63 (60.0%) | 0.652 |
| Dyspnea | 5 (16.1%) | 15 (14.3%) | 0.800 |
| Blood work |  |  |  |
| CRP, mg/l; median (IQR) | 93.0 (54.9, 148.5) | 135.0 (65.7, 226.0) | 0.021 |
| WBC, x 10^9/l; median (IQR) | 13.1 (10.9, 18.1) | 17.8 (12.1, 24.5) | 0.010 |
| ANC, x 10^9/l; median (IQR) | 7.7 (5.9, 10.3) | 10.7 (6.4, 17.2) | 0.032 |
| Chest x-ray; n (%) | 24 (77.4%) | 85 (81.0%) | 0.666 |
| Microbiology; n (%) |  |  |  |
| Adenovirus | 6 (19.4%) | 18 (17.1%) | 0.777 |
| Influenza (A/B) | 3 (9.7%) | 2 (1.9%) | 0.044 |
| Rhino-/Enteroviruses | 4 (12.9%) | 10 (9.5%) | 0.588 |
| RSV | 5 (16.1%) | 7 (6.7%) | 0.104 |
| Admission rate, n (%) | 29 (93.5%) | 96 (91.4%) | 0.705 |
| Length of stay, d; mean (SD) | 2.6 (1.2) | 3.4 (1.8) | 0.022 |
| Discharge diagnosis group; n (%) |  |  |  |
| LRTI | 6 (19.4%) | 52 (49.5%) | 0.003 |
| URTI | 20 (64.5%) | 35 (33.3%) | 0.002 |
| Non-RTI | 5 (16.1%) | 18 (17.1%) | 0.895 |

IQR, Interquartile range; SD, standard deviation; CRP, C-reactive protein; WBC, white blood count; ANC, absolute neutrophil count; MMBV, MeMed BV; RTI, respiratory tract infection; RSV, respiratory syncytial virus; LRTI, lower respiratory tract infection; URTI, upper respiratory tract infection.

p-values were calculated using the Mann-Whitney U test for ‘age’, ‘time from symptoms onset’, ‘length of stay’, ‘temperature’ and blood work variables. The remaining p-values were calculated using Richardson's method.

Discharge diagnoses were coded using medDRA classification system; a full list is provided in supplementary table 1 and how they are grouped into LRTI and URTI is described in supplementary table 2.
